# Movement Path Data Generation from Wi-Fi Fingerprints for Recurrent Neural Networks

**DOI:** 10.3390/s21082823

**Published:** 2021-04-16

**Authors:** Hong-Gi Shin, Yong-Hoon Choi, Chang-Pyo Yoon

**Affiliations:** 1 NEOWIZ Corp. 14, Daewangpangyo-ro 645beon-gil, Bundang-gu, Seongnam-si 13487, Gyeonggi-do, Korea; ghdrl95@neowiz.com; 2 School of Robotics, Kwangwoon University, 20, Kwangwoon-ro, Nowon-gu, Seoul 01897, Korea; 3 Department of Computer & Mobile Convergence, Gyeonggi University of Science and Technology, 269, Gyeonggigwagidae-ro, Siheung-si 15073, Gyeonggi-do, Korea

**Keywords:** deep learning, data pre-processing, Wi-Fi fingerprint, recurrent neural network, K-means clustering

## Abstract

The recurrent neural network (RNN) model, which is a deep-learning network that can memorize past information, is used in this paper to memorize continuous movements in indoor positioning to reduce positioning error. To use an RNN model in Wi-Fi-fingerprint based indoor positioning, data set must be sequential. However, Wi-Fi fingerprinting only saves the received signal strength indicator for a location, so it cannot be used as RNN data. For this reason, we propose a movement path data generation technique that generates data for an RNN model for sequential positioning from Wi-Fi fingerprint data. Movement path data can be generated by creating an adjacency list for Wi-Fi fingerprint location points. However, creating an adjacency matrix for all location points requires a large amount of computation. This problem is solved by dividing indoor environment by K-means clustering and creating a cluster transition matrix based on the center of each cluster.

## 1. Introduction

Recently, Wi-Fi fingerprinting has been used to construct indoor positioning systems [1,2,3,4,5,6,7,8]. In this approach, the Wi-Fi fingerprinting system records the received signal strength indicator (RSSI) of the access points (APs) collected at each location point in the database. It then compares input data with the recorded data to calculate a position. The signal strength values of Wi-Fi APs can lead to incorrect positions because they contain noise caused by obstacles. To improve the location accuracy and reduce the adverse effects of environmental factors, several types of studies have been conducted. A method of fingerprint location for Wi-Fi signals assisted by smart phone built-in sensors has been studied in work [9]. To improve the location accuracy, Wang et al. [10] utilized 5G mmWave beam. However, these approaches require auxiliary devices, which makes configuration and operation complex.

Another approach to improve location accuracy is to use RSSI with mobile user trajectory [11,12,13]. An approach for fusion of dead reckoning trajectories generated from foot-mounted inertial measurement units (IMUs), RSSI from Wi-Fi signals and position estimations from global positioning system (GPS) from multiple users was proposed for trajectory estimation and crowd-sourced RM generation [12]. A Wi-Fi RSSI dataset containing sequentially collected trajectories at a finer level of reference point is presented in [13]. Positioning accuracy can be improved by using a dataset containing trajectory, however, it is difficult to apply in a real environment because it takes a lot of effort to prepare and process the dataset. Also, there is a limitation that only the movement paths followed during the collection process exist as a dataset.

It is often difficult to estimate a movement path of mobile users from a dataset (e.g., collected Wi-Fi fingerprint) for which trajectory is not provided. Regarding the movement path generation, recurrent neural network (RNN) models capable of learning time series information are attracting attention. It is well-known that the RNN can extract features from high-dimensional time series input data and perform well in classification and regression problems. The RNN model is a supervised learning algorithm that can consider the continuity of the data [14,15,16]. This network can consider the continuous movement of a person in indoor positioning and hence calculate his or her current position more accurately or predict his or her next movement. To use an RNN in an indoor positioning system, RSSIs must be sequentially input according to a human’s path. However, most of the datasets provided for indoor positioning studies do not provide this path.

This paper proposes a method to generate movement path data based on Wi-Fi fingerprinting. We use K-means clustering to create clusters that separate indoor location areas. The generated clusters and an adjacency matrix for these clusters are used to create path data by converting them into the states and state transition probabilities of a Markov chain. The proposed machine learning model uses the generated movement path data instead of Wi-Fi fingerprinting, so the RSSIs of the previous location affect the positioning of the current location. We compared the performance of the proposed technique with that of the Wi-Fi fingerprint-based positioning method.

The structure of this paper is as follows. Section 2 describes K-means clustering and Markov chains. Section 3 describes methods to divide an indoor environment based on K-means clustering and generate movement path data. Section 4 describes the performance evaluation of the proposed and existing positioning algorithms. Finally, Section 5 presents the conclusion and future work of this study.

## 2. Related Works

### 2.1. K-means Clustering

K-means clustering is an algorithm that classifies given data into *K* clusters. It updates the centroid of cluster in a way that minimizes the variance of the distance between clusters. Assume that all data in set D belongs to one of the clusters in set C. When $D=C_{1}\cup C_{2}\ldots \;\cup \;C_{K},\;C_{i}\cup C_{j}=\emptyset$ and the number of clusters is K, the cluster to which data point $d_{j}$ belongs is calculated as follows:

(1) $${\mathrm{argmin}}_{c}\overset{K}{\underset{i=1}{\sum }}\underset{d_{j}\in C_{i}}{\sum }\;\|d_{j}-c_{i}\|{}^{2}$$

Algorithm 1 shows the K-means clustering algorithm used in this paper. It takes D, K, and the maximum number of iterations as input, and outputs cluster center set C and cluster index set L, which indicates to which cluster data point $d_{i}$ belongs. The center points of a cluster are initialized using the method described in the K-means++ algorithm [17].


**Algorithm 1 K-means Clustering Algorithm**
**Input:**$D=\left\{d_{1},\;d_{2},\;\ldots ,\;d_{n}\right\}$ /* set of data to be clustered */   K /* number of clusters */   M /* limit of iterations */**Output:**$C=\left\{c_{1},\;c_{2},\;\ldots ,\;c_{K}\right\}$ /* set of cluster centroids */    $L=\left\{l\left(d_{i}\right)|i=1,\;\ldots ,n\right\}$ /* set of cluster labels of *D* */
**begin**
 C initialized by K-means++; **for each**
$d_{i}\in D$
**do**  $l\left(d_{i}\right)\leftarrow {\mathrm{argmin}}_{j\in \left\{1,\ldots ,K\right\}}d\left(d_{i},\;c_{j}\right)$; **end** chagned←false; iter←0; **repeat**  **for each**
$c_{i}\in C$
**do**    UpdateCluster($c_{i}$);   **end**   **for each**
$d_{i}\in D$
**do**    $minDist\leftarrow {\mathrm{argmin}}_{j\in \left\{1,\ldots ,K\right\}}d\left(d_{i},\;c_{j}\right)$;    **if**
$minDist\neq l\left(d_{i}\right)$
**then**     $l\left(d_{i}\right)\leftarrow minDist$;     changed←true;    **end**   **end**   iter←iter+1;  **until**
changed=true and iter≤M;
**end**


### 2.2. Markov Chains

The Markov property states that the current state is affected by the past state. Markov chains are discrete probability processes with Markov properties and change state at every time step. In a Markov chain, the probability of transitioning to another state is expressed by the state transition probability matrix. Assuming states C=1, 2, 3, …, K*,* the state transition matrix is represented as follows:

(2) $$P=\left[\begin{array}{ccc} \begin{array}{ccc} p_{11} & p_{12} & p_{13}\\ p_{21} & p_{22} & p_{23}\\ p_{31} & p_{23} & p_{33} \end{array} & \cdots & \begin{array}{c} p_{1K}\\ p_{2K}\\ p_{3K} \end{array}\\ \vdots & \ddots & \vdots \\ \begin{array}{ccc} p_{K1} & p_{K2} & p_{K3} \end{array} & \cdots & p_{\mathrm{KK}} \end{array}\right]$$

where $p_{ij}\geq 0$ and for all i, the following formula holds:

(3) $$\overset{K}{\underset{j=1}{\sum }}p_{ij}=\overset{K}{\underset{j=1}{\sum }}P(C_{t+1}=j\left|C_{t}=i)\right.=1$$

### 2.3. Fingerprint Positioning Technique

The positioning algorithm compares the input data with the radio fingerprint and estimates it as the most similar location. Euclidean distance is representative distance comparison algorithm, but the distance difference becomes ambiguous when high-dimensional data is input [1]. To solve this problem, Shrestha studied logarithmic Gaussian distance, which shows high performance in high-dimensional data [3]. Tian studied affinity propagation clustering, which selects clusters with features like input data and compares Euclidean distances within the cluster [5]. Positioning algorithms using deep neural networks (DNN) have been studied to use higher-dimensional input data. Zhang improved the positioning accuracy by designing a layer that mixed DNN and hidden Markov model (HMM) [6]. Park studied data augmentation techniques for generating Wi-Fi fingerprints with high density data and parallel learning for learning multistory buildings [4]. Sahar and Han collected Wi-Fi fingerprints by walking survey dataset and used them as input to the LSTM model [2].

## 3. Proposed Method

### 3.1. Clustering of Location Points

Dataset must be collected by transitioning to adjacent location points over time to generate movement path data based on Wi-Fi fingerprinting. Adjacency lists that contain the adjacent location points for all location points require a large amount of computation to generate according to dataset size. In addition, this approach may generate movement paths with non-mobility data or long distances data that cannot be moved by humans depending on the density of the location points.

To solve this problem, we create clusters based on the set of location point in the Wi-Fi fingerprint data to separate indoor areas. The K-means clustering algorithm shown in Algorithm 2 computes the centroids of the set of clusters *C* for the location points of Wi-Fi fingerprint *D*. This study does not consider multistory buildings, so the centroid of each cluster $c_{i}$ stores the X-axis and Y-axis data. The centroids of cluster set C use the adjacency matrix:

(4) $$A=\left[\begin{array}{ccc} \begin{array}{ccc} a_{11} & a_{12} & a_{13}\\ a_{21} & a_{22} & a_{23}\\ a_{31} & a_{23} & a_{33} \end{array} & \cdots & \begin{array}{c} a_{1K}\\ a_{2K}\\ a_{3K} \end{array}\\ \vdots & \ddots & \vdots \\ \begin{array}{ccc} a_{K1} & a_{K2} & a_{K3} \end{array} & \cdots & a_{\mathrm{KK}} \end{array}\right]$$

where $a_{ij}$ is computed as follows:

(5) $$a_{ij}=\left\{\begin{array}{c} 1,\;\sqrt{{\left(x_{i}-x_{j}\right)}^{2}+{\left(y_{i}-y_{j}\right)}^{2}}\;\leq d_{max}\\ 0,\;\;\;\;\;\;\;\;\;\;\;\;\;\;\;\;\;\;\;\;\;\;\;\;\;\;\;\;\;\;\;\;\;\;\;\;\;\;\;\;\;\;\;\;otherwise\; \end{array},\right.$$

where $a_{ij}$ stores the adjacency of $c_{i}$ and $c_{j}$, coordinate $\left(x_{i},\;y_{i}\right)$ is centroid positions of $c_{i}$, coordinate $\left(x_{j},\;y_{j}\right)$ is centroid positions of $c_{j}$, and $d_{max}$ is the maximum distance. If the distance between $c_{i}$ and all the other clusters is larger than this distance, $c_{i}$ is merged with the nearest cluster.

The adjacency list $a_{i}=\left[a_{i1},\;a_{i2},\;\ldots ,a_{iK}\right]$ represents how a cluster can move from cluster $c_{i}$. Because the cluster selected at the current time is affected by the cluster selected at the previous time, the clusters can be expressed as a Markov chain. The probability of transition between clusters is expressed by the state transition probability matrix P of the Markov chain as follows:

(6) $$p_{ij}=\left\{\begin{array}{c} \frac{1}{\sum_{k=1}^{K}a_{ik}},\;a_{ik}=1\\ 0,\;\;\;\;\;\;\;\;\;\;otherwise \end{array}\right.$$

where $p_{ij}$ is the transition probability of moving from $c_{i}$ to $c_{j}$. In this paper, we do not consider movement frequencies for the positioning environment, so the transition probability is equal for all clusters adjacent to $c_{i}$. Algorithm 2 shows the proposed state transition matrix initialization algorithm for the clusters. The state transition matrix P for a cluster does not need to be changed unless the structure of the indoor environment changes.


**Algorithm 2 Cluster State Transition Matrix Generation Algorithm**
**Input:**$C==\left\{c_{1},\;c_{2},\;\ldots ,\;c_{K}\right\}$ /* set of cluster centroids */   K /* number of clusters */   $d_{max}$ /* distance limit */**Output:**$P=\left\{p_{11},\;\ldots ,\;p_{KK}\right\}$ /* set of cluster transition matrix */
**for**
i←1
**to**
K
**do**
  $c_{min}\leftarrow i$;  $d_{min}\leftarrow 0$;  flag←false;  **for**
j←1
**to**
K
**do**    $d\leftarrow \mathrm{distance}\left(c_{i},\;c_{j}\right)$;    **if**
i=j
**or**
$d\leq d_{max}$
**then**     $a_{ij}\leftarrow 1$;     flag←true;    **else**     $a_{ij}\leftarrow 0$;     **if**
$c_{min}=i$ or $d_{min}\geq d$
**then**      $c_{min}\leftarrow j$;      $d_{min}\leftarrow d$;     **end**    **end**   **end**   **if**
flag=false
**then**    $j\leftarrow c_{min}$;    $a_{ij}\leftarrow 1$;  **end**
**end**

**for**
i←1
**to**
K
**do**
  $near\_num\leftarrow \sum_{j=1}^{K}a_{ij}$;  **for**
j←1
**to**
K
**do**   **if**
$a_{ij}=1$
**then**    $p_{ij}\leftarrow \frac{1}{near\_num}$;   **else**    $p_{ij}\leftarrow 0$;   **end** **end**
**end**


### 3.2. Generation of Movement Path Data Using Clustered Fingerprint Data

**Figure 1:**
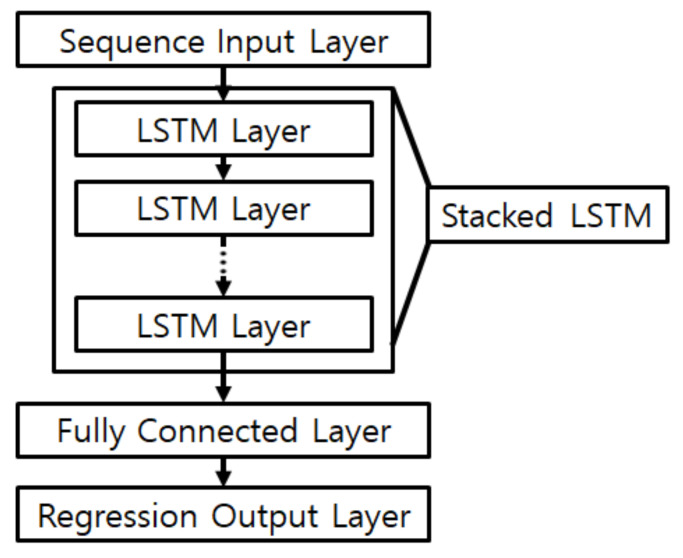
Architecture of the proposed neural network for movement path data.

This section proposes a method for creating movement path data using cluster labels L and cluster transition matrix P of the data calculated by Algorithms 1 and 2. This paper does not consider indoor structures and does not specify start and end clusters. For this reason, the path is created by traversing clusters according to the path length $path_{max}$ from a randomly chosen cluster [18]. The movement path randomly extracts one data point in the current cluster to generate input data and uses the location point of the last visited cluster as a label. Because the input size of the learning model is proportional to $path_{max}$, not only does the amount of computation increase, but so does the amount of old data that is not needed to predict the current location. Hence, $path_{max}$ considers a time interval over which the RSSI and device performance is collected. The generated movement path data consists of RSSI data for each AP over time, so a long short-term memory (LSTM) layer for time-based data can be used, as shown in Figure 1.

The size of the input layer in Algorithm 1 is the number of APs in the Wi-Fi fingerprinting system multiplied by $path_{max}$. The learning model predicts the location using the last result of the LSTM layer as an input to the fully connected layer. Algorithm 3 shows the movement path data generation algorithm proposed in this paper. The algorithm input consists of dataset D and cluster labels L of the data. The Wi-Fi fingerprint training and testing sets are input separately. If the amount of data in the Wi-Fi fingerprint is small, there is a high probability that duplicate data will be generated. Therefore, an appropriate value for m should be used.


**Algorithm 3 Movement Path Generation Algorithm**
**Input: **$D=\left\{d_{1},{\;d}_{2},\;\ldots ,{\;d}_{n}\right\}$ /* set of data to be clustered */    $L=\left\{l\left(d_{i}\right)\left|d=1,\;\ldots ,\;n\right.\right\}$ /* set of cluster labels of *D* */    $P=\left\{p_{11},\;\ldots ,{\;p}_{\mathrm{KK}}\right\}$ /* set of cluster transition matrix */    ${\mathrm{path}}_{\max}$ /* maximum path length */    m /* number of data to generate */    K /* number of clusters */    $d_{\max}$ /* distance limit */**Output:**$\;T=\left\{t_{1},\;\ldots ,{\;t}_{m}\right\}$ /* set of sequential location data */
**for**
 iter←1
**to**
m
**do**
  $i\leftarrow k\in \left\{1,\;2,\ldots ,\;K\right\}$;  $t_{\mathrm{iter}}\leftarrow d_{i}\in D$ for all
$\;l\left(d_{i}\right)=i$;
**for**
 path←2
**to**
${\mathrm{path}}_{\max}$
**do**
   i←$k\in \{1,\;2,\ldots ,\;K|p_{i}\}$;   $t_{\mathrm{iter}}\leftarrow \mathrm{concat}(t_{\mathrm{iter}},{\;d}_{i}\in D$$\;\mathrm{for}\;\mathrm{all}\;l\left({\;d}_{i}\right)=i)$;  **end**
**end**


## 4. Experiment

### 4.1. Experimental Environment

We use a published Wi-Fi fingerprint dataset to compare the performance of the proposed method with existing methods. The dataset consists of RSSI fingerprint collected from 21 devices installed at the Tampere University of Technology (TUT) in Finland [19]. As shown in Table 1, the number of training data in the TUT dataset is smaller than the number of test data. The training and test sets of the TUT dataset were exchanged to increase the amount of training data. In addition, this study does not consider multistory buildings, so only the ground floor data in the TUT dataset were used.

### 4.2. Experimental Scenario

The experiment compares the positioning performance of the proposed method and that of the existing methods 3-Layer DNN, P-DNN [4], DNN-DLB [20], 2D-CNN-DLB [20], RSS clustering [21], and 3D clustering [21]. The existing methods determine the position using the Wi-Fi fingerprint inputs. The proposed method determines the position using movement path data generated from the Wi-Fi fingerprints. K-means clustering was used to create clusters from the training and test sets of the TUT dataset. The state transition matrix initialization algorithm was used to create the state transition probability of the clusters, and the movement path data generation algorithm was used to generate the movement path data for learning and testing with state transition probabilities, training set, and test set.

**Figure 2:**
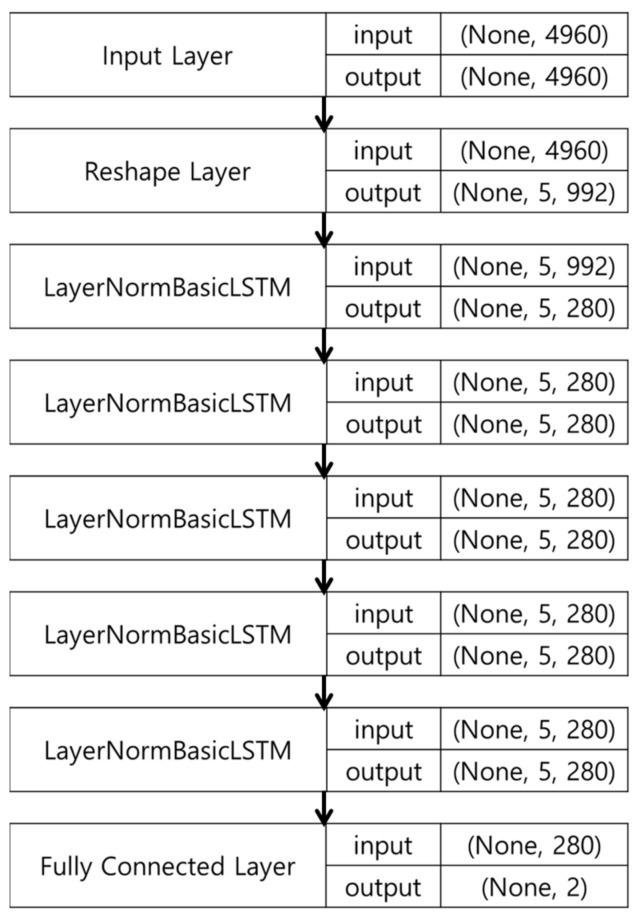
Proposed deep learning layer at p=5

The architecture of a layer of the RNN model used in the proposed technique is shown in Figure 2 for *p* = 5. The input layer was set to $992\times path_{max}$ and the output layer was used to regress the positioning coordinates X and Y.

The hyperparameters of the K-means clustering and path data generation method are shown in Table 2. A Bayesian optimizer was used to optimize hyperparameters [22]. This study used the search range listed in Table 3 to find the optimal hyperparameter settings.

### 4.3. Experimental Results

**Figure 3:**
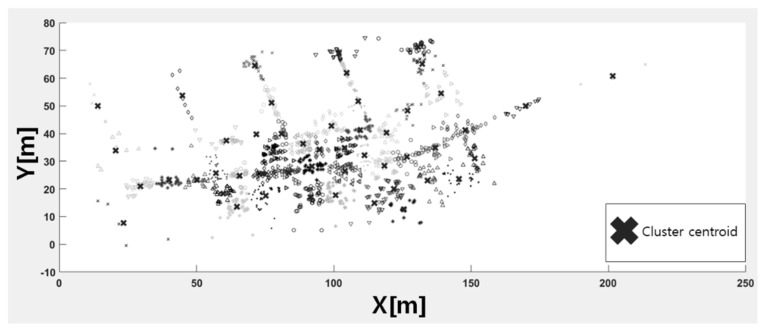
K-means clustering result on TUT Wi-Fi fingerprint dataset (K = 50).

Figure 3 shows the results of applying the location points of the TUT Wi-Fi fingerprint dataset to the K-means clustering of Algorithm 1. In Figure 3, the X marks the center of the cluster and the other symbols indicate the location of data point. To distinguish the cluster to which the data point belongs, when marking the data point, the symbol (e.g., triangle, plus signal, circle, etc.) and color are expressed differently. The sum of the distances between the centers of the clusters and the observation points averages 438.36 m, with a minimum distance of 52.66 m and a maximum distance of 972.19 m.

**Figure 4:**
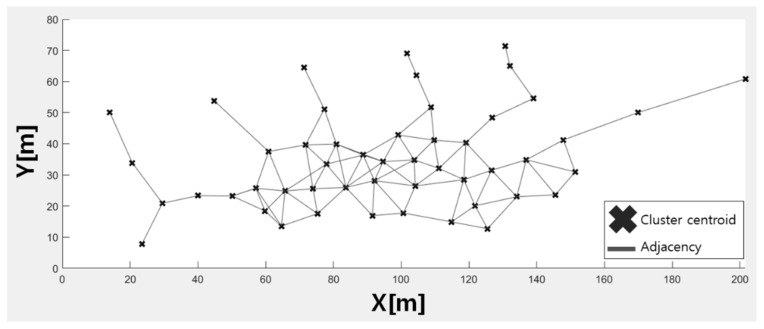
Result of creating neighbor list of clusters $\;\left(d_{max}=15\right)$

Figure 4 shows the result of generating a neighbor list between midpoints of a cluster. The cluster on the right side of the figure is connected to the nearest cluster because the distance to all clusters is greater than the distance limit. This paper does not consider the building structure, however it can generate a neighbor list similar to that of the TUT dataset.

Table 4 shows the mean error of each method for the TUT dataset. The results show that proposed method yields the lowest average error. The movement path data includes various paths to reach the same position. Therefore, it can be concluded that the positioning is accurate because the previous RSSI influences the current RSSI.

## 5. Conclusions

In this paper, we proposed a method to generate movement path data based on information gathered from Wi-Fi RSSI. This data is used as input data for an RNN model to reduce the position error of an indoor positioning system. The method used to generate movement path data is based on K-means clustering and Markov chains. Since the Wi-Fi fingerprint dataset did not include time information, we had to use a single set of RSSIs as input for machine learning. To solve this problem, we divided the location points of the Wi-Fi fingerprinting area into clusters and created movement paths for discrete time steps based on a Markov chain. The experimental results of the proposed technique on the TUT dataset yielded an average error of about 4.9 m, which is lower than that of other existing methods.

The proposed method generated data through a random walk without considering a multi-level indoor structure. This approach can generate a movement path for an open indoor space, but it cannot generate movement paths for a unidirectional movement space (e.g., a museum or aquarium).

## Figures and Tables

**Table 1:** TUT Wi-Fi fingerprint dataset about ground floor.

Name	Value
Area size	108 m ×208 m
Number of training data	697
Number of test data	3951
Number of APs	992

**Table 2:** Hyperparameters for movement path data generation.

Hyperparameter	Value
Number of clusters *k*	50
Maximum number of iterations *M*	1000
Distance limit $d_{max}$	15m
Path *p*	5
$\mathrm{Number}\;\mathrm{of}\;\mathrm{generated}\;\mathrm{training}\;\mathrm{data}\;m_{train}$	20,000
$\mathrm{Number}\;\mathrm{of}\;\mathrm{generated}\;\mathrm{test}\;\mathrm{data}\;m_{test}$	5000

**Table 3:** Search ranges for hyperparameter optimization.

Hyperparameter	Value
Minibatch size	50 (fixed)
Learning rate	0.001—0.05
Dropout	0.5—1
Number of stacked LSTMs	2—7
Number of LSTM hidden cells	100—Input size × 2
Number of epochs	20—1000

**Table 4:** Mean error of positioning algorithms on the TUT dataset.

Algorithm	Mean Error [m]
2-Layer LSTM with movement path data (proposed)	4.91
DNN-DLB [20]	5.33
3-Layer DNN	5.73
Data Augmented 5-Layer P-DNN [4]	6.94
2D-CNN-DLB [20]	7.08
RSS clustering (affinity propagation) [21]	8.08
3D clustering (K-means) [21]	14.80

## Data Availability

The data presented in this study are available on request from the authors.
